# Photo induced reaction of myoglobins with energy transferred from excited free tryptophan†

**DOI:** 10.1039/d0ra09341f

**Published:** 2020-12-09

**Authors:** Hong-Yu Cao, Yu-Qi Ma, Ling-Xing Gao, Qian Tang, Xue-Fang Zheng

**Affiliations:** College of Life Science and Biotechnology, Dalian University Dalian 116622 China caohongyu@foxmail.com; College of Environmental and Chemical Engineering, Dalian University Dalian 116622 China dlxfzheng@126.com; Liaoning Key Laboratory of Bio-Organic Chemistry, Dalian University Dalian 116622 China

## Abstract

Despite being one of the most studied proteins in biology, the photolysis mechanism of myoglobin heme affected by endogenous substances free amino acids is still in controversy. The transient absorption and kinetic processes of photo-excited myoglobin in three forms and the effects of free excited tryptophan on redox reaction of myoglobin were monitored by laser flash photolysis. With dual energy superposition of direct light irradiation and indirect energy transferred from the free excited tryptophan, the variation value in optical density (ΔOD) of MetMb increased by 66.7%, from 0.9 to 1.5. The ΔOD value of MbO_2_ in ferrous form increased from 0.9 to 1.25, while the ΔOD value of DeoxyMb increased from 0.75 to 1.2. The decay time of excited DeoxyMb was prolonged obviously with the excited tryptophan, while the decay time of excited MbO_2_ and MetMb was shortened significantly. The excited tryptophan could promote laser induced reaction processes of myoglobin in different forms by intermolecular energy transfer to one final similar photo reaction state. The possible photo induced reaction mechanisms of DeoxyMb, MbO_2_, MetMb with and without free tryptophan were also proposed.

## Abstract 2

The dual energy superposition of direct light irradiation and indirect energy transferred from the free excited tryptophan promoted the photo induced reaction of myoglobin.

## Graphical abstract

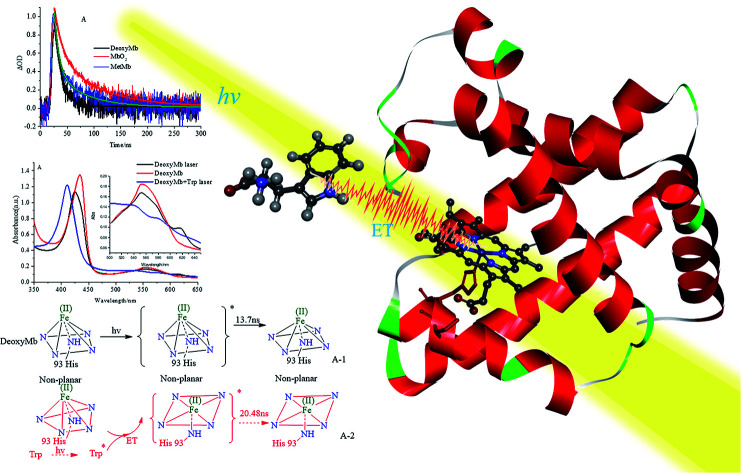

## 1. Introduction

Myoglobin (Mb), an oxygen-binding protein found in the muscle tissue of vertebrates in general and in almost all mammals,^1^ contains a porphyrin ring with an iron at its centre. A proximal histidine group (His-93) coordinates directly to the iron, and a distal histidine group (His-64) hovers near the opposite porphyrin plane.^2,3^ The iron in the myoglobin can reversibly bind with small molecular ligands, such as O_2_, CO and NO. The tight sequestration of iron within myoglobin serves two protective functions which can be classified as chemical and biological.^4^ Several experiments proved that the ferric state myoglobin could return to ferrous state with ultraviolet light irradiation.^5,6^ The ultrafast reaction process of myoglobin induced by laser has been studied since the late 1980s.^7–11^ Proteins showed considerable changes after electron beam irradiation in terms of structure.^12^ The growing consensus that the photo excited porphyrin will provide electron transfer from porphyrin ring to iron d-orbital^13–16^ is nearly reached, but the photolysis mechanism of the diatomic ligands is still in dispute. So, the binding kinetics and laser flash photolysis of ligands have attracted many researchers' interest. Time-resolved spectroscopic measurements after photodissociation of the ligand revealed a complex ligand-binding reaction with multiple kinetic intermediates, resulting from protein relaxation and movements of the ligand within the protein.^17,18^ Hochstrasser *et al.*^9^ detected that the kinetics of the spectral evolution of myoglobin following the Soret band excitation (405 nm) were measured in the Q-band (450–630 nm) and band-III (730–900 nm) regions. The results discovered that the rotation of His-64 was identified as the characteristic behaviour of a transient species produced by myoglobin-CO photolysis. Photo-dissociation of CO initiated by the 532 nm output from Nd:YAG laser indicated that the photolysis of the CO–Fe bond and the subsequent ligand escape from the protein matrix into surrounding solvent is fast (*τ* < 50 ns).^19^ F43Y Mb mutant with a tryptophan (Trp)–heme cross-link can efficiently catalyse the oxidation of indole to indigo, which proved the significant effects of Trp on myoglobin functions.^20^

The optical properties of tryptophan (Trp),^21,22^ tyrosine (Tyr),^23^ phenylalanine (Phe)^24^ and cysteine (Cys)^22^ are widely applied in the determination of protein spectrum, especially in the region of near ultraviolet. Hayon *et al.*,^21^ using laser flash photolysis, showed that the photo-excited tryptophan transformed to a single excited tryptophan accompanied by two non-radiative relaxation pathways. Electron (e_aq_^−^) and tryptophan cationic free radicals Trp˙^+^ are generated firstly, and then Trp˙^+^ is rapidly converted into Trp˙ and H^+^ ((1) and (2)). The triplet formation ^3^Trp is generated by intersystem crossing from the photo excited state of singlet formation ^1^Trp* ((3) and (4)). The molecular energies of various excited tryptophan states are rather high and easy to transfer among molecules in the solution.

1 Trp + *hv* → Trp˙^+^ + e_aq_^−^

2 Trp˙^+^ → Trp˙ + H^+^

3 ^1^Trp + *hv* → ^1^Trp*

4 ^1^Trp* → ^3^Trp

Porphyrin in myoglobin has a large conjugated system, and its absorption wavelength range becomes wider when the transition metal iron with d orbital coordinate to the porphyrin. Ultrafast two-dimensional ultraviolet spectroscopy experiments have determined the electron transfer and excitation energy transfer rates of the two intrinsic tryptophan to heme in myoglobin.^25^ The theorical results demonstrate the small changes in the distance and the orientation of the inner tryptophan residues relative to the heme, have a large effect on tryptophan–heme electron and excitation energy transfer rates.^26^ The native Phe, Tyr, and Trp concealed in the inner myoglobin and were far from iron porphyrin moiety in the protein, hence they were difficult to achieve the ideal excited states and complete the energy transfer processes for the obstruction by other amino acids. In our previous research works, we found that both visible light and UV light^5,27^ irradiation could promote oxidation of ferroporphyrin, which significantly affected the physiological function of oxymyoglobin (MbO_2_). The electron transfer from Trp to heme in ferrous myoglobin was proved and the reduction generates a low valence Fe-porphyrin anion radical.^28^ Based on the experiments, we proposed that free Phe, Tyr and Trp can participate the light oxidation reaction.^5^ Our previous study verified that the free endogenous substance tryptophan could change photo-reduction process of cytochrome c,^6^ while later experiments confirmed that Phe, Tyr and Trp could promote the photo reaction of Cyt c.^27^

Based on the former conclusion we realized that some of the endogenous substances could affect bio-macromolecules in various undiscovered manners. Hence, we proposed that as a good energy and electron acceptor, the heme moiety can absorb the energy or electrons released from the excited free tryptophan in solution. The further investigations including transient absorption and kinetic process of photo-excited myoglobin should be conducted urgently. The effects of free Trp on redox reaction of myoglobin induced by laser are also lucubrated with spectroscopic methods. The preliminary reaction mechanisms are proposed based on a variety of spectral data.

## 2. Materials and methods

The UV-Vis absorption spectra showed that the horse heart myoglobin (purchased from Sigma, USA) was in met-myoglobins (MetMb) state. The MetMb was dissolved in Na_2_HPO_4_–NaH_2_PO_4_ (PB) buffer (0.05 mol L^−1^, pH = 7.4). Tryptophan was dissolved into 0.05 mol L^−1^ PB buffer solution with a concentration of 0.01 mol L^−1^. To prepare the DeoxyMb, MetMb was reduced with proper amount of sodium hyposulfite (0.1 mol L^−1^) and purged high-purity N_2_ for 0.5 h. To obtain MbO_2_, the MetMb was reduced with sodium hyposulfite (0.1 mol L^−1^) and purged high-purity O_2_ for 0.5 h. The concentration and purity of DeoxyMb and MbO_2_ were determined by UV absorption. All the final testing concentrations of myoglobins and free tryptophan were 10^−5^ mol L^−1^ in the cuvette. The water used in the experiment was all deionized water.

The UV-Vis absorbance spectra were measured in a 1 cm quartz cell and recorded using UV-Vis spectrophotometer (V-560, Jasco). The circular dichroism (CD) spectra were measured by CD spectroscopy (J-810, Jasco). The scanning speed of CD spectra was 50 nm min^−1^ with 1 nm band width and 2 s response time. A total of accumulations 3 were collected to obtain the available data.

The photolysis experiments were conducted using a laser flash photolysis spectrometer (LP980, Edinburgh Instruments, UK), in combination with a flash lamp pumped Q-switched Nd:YAG laser. A 266 nm laser pulse was focused onto the 1 cm sample cuvette to trigger the photo excited reactions of myoglobin and the detection light from a xenon lamp passed the myoglobin at vertical angle to the path of the exciting pulse. Transient species signals are detected by Hamamatsu R928 photomultiplier tube. The transient absorption spectrum acquisition time was 1000 ns, while the wavelength range was 250–800 nm and the number of slices was set to 110 (wavelength width: 5 nm). The change in the sample transmission allows the change in absorption to be calculated, typically in units of optical density, ΔOD(*t*, *λ*) (5):5
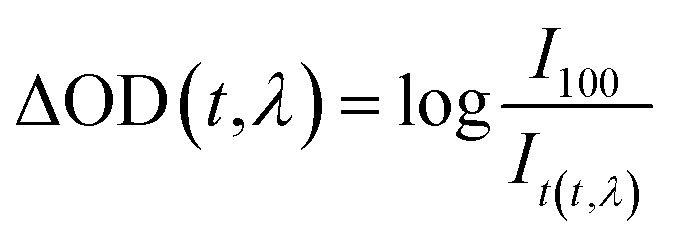
*I*_100_ is the light level measured through the sample before excited states are created.

The decay kinetics curves starting time are calculated from the time of maximum absorption values. The exponential decay process was expressed in mathematical terms as eqn (6):6
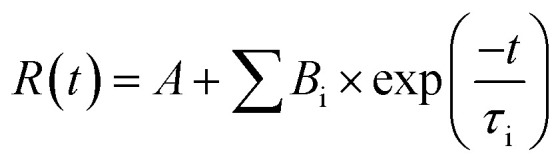
with pre-exponential factors *B*_i_, the characteristic lifetimes *τ*_i_ and an additional background *A*. Here *R*(*t*) is called the sample decay model of ΔOD(*t*). If ΔOD(*t*) curves are being analysed, then the *B*_i_ values represent the optical density at time zero. The numerical procedure behind the search for the best *B*_i_ and *τ*_i_ is the iteration procedure Levenberg Marquardt algorithm (7).7
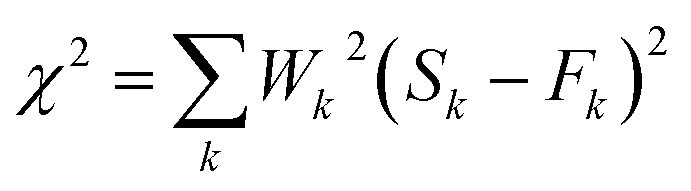
where *F*_*k*_ are the raw measured data and *S*_*k*_ is the data points of the fitted curve. *k* is the index for the individual data points to be fitted, the sum expands over all these data points. The fitting exponential equation was determined by *χ*^2^ and standard deviation (std. dev.).

## 3. Results and discussions

### 3.1 Laser flash photolysis transient absorption spectra and kinetics curves of myoglobin

**Fig. 1:**
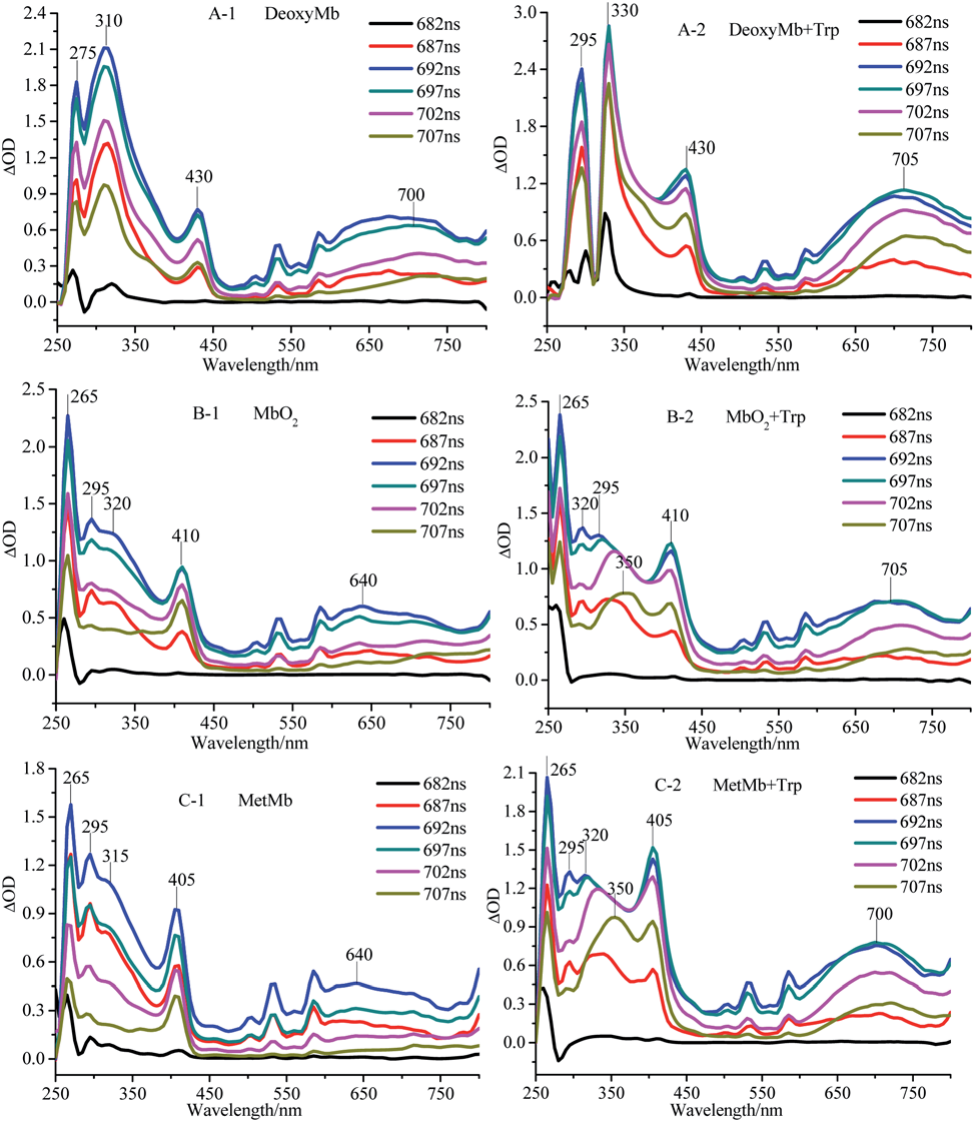
Transient absorption spectra of DeoxyMb (A), MbO_2_ (B) and MetMb (C) irradiated by 266 nm laser. The concentrations of Mb and free Trp were 10^−5^ mol L^−1^.

The transient absorption spectra of DeoxyMb, MbO_2_ and MetMb protein solution irradiated by 266 nm laser at different moments were shown in Fig. 1. The absorption peaks at 265 and 295 nm were the bleaching peaks, while the absorption peaks at 310, 315 or 320 nm were the absorption peaks of the excited tryptophan in the protein. The absorption peak at 430 nm was the character of ferrous myoglobin, while the peak at 410 nm verified MbO_2_. The absorption peak 405 nm proved the existence of ferric myoglobin. Further analysis of the transient absorption values at different wavelengths with time revealed that the variation trend of the wavelengths in the absorption band kept similar. The intensity of absorption peaks increases first and then decreases with the time lapse. At the time of 697 ns after photo-irradiated moment, the maximum intensity of absorption peak is observed in the spectrum.

The maximum transient absorption peaks of Trp irradiated by 266 nm laser located at 320, 340 and 695 nm. The peak at 695 nm was attributed to electron absorption of hydrated Trp, while peaks at 320, 340 nm were assigned to the absorption of Trp˙ and Trp˙^+^,^21,22^ respectively. The transient absorption spectra of myoglobin in three states with free tryptophan revealed that the absorption peaks of iron porphyrin remain in the same places (Fig. 1), but the absorption peak intensities increased obviously, which manifested the energy transfer between the excited tryptophan and protein heme. The superposition of self-absorption light energy and tryptophan transferred energy enriched the electron transition types of the large conjugated system, resulting in the increase of the absorption peak intensities of iron porphyrin.

The absorption peak of excited Trp in DeoxyMb solution red-shifted from 310 nm to 330 nm, while intensity of the peak decreased gradually with the passage of time. The phenomenon might be ascribed to the energy transfer from the excited Trp to the iron porphyrin of DeoxyMb induced the valence state of iron in the protein. The maximum absorption wavelengths of free Trp gradually red shifted to 350 nm with the decrease of intensities, resulting in a variety of Trp transients forms and hydrated peaks at 700 nm. It is shown that the excited free tryptophan transferred energy to MbO_2_ and MetMb, while the excited myoglobins returns to the ground state.

With the energy transferred from free Trp, the UV absorption intensities of porphyrins in three myoglobin states increased obviously. In MetMb, the value of ΔOD intensity increase by 66.7%, from 0.9 to 1.5. The ΔOD value of MbO_2_ in ferrous form increased from 0.9 to 1.25, while the ΔOD value of DeoxyMb increased from 0.75 to 1.2. The ΔOD values of MbO_2_ and DeoxyMb increased by 38.8% and 60%, respectively. The above results revealed that the contributions of native Phe, Tyr, and Trp in myoglobin were lower for the reaction than that of free Trp.

**Fig. 2:**
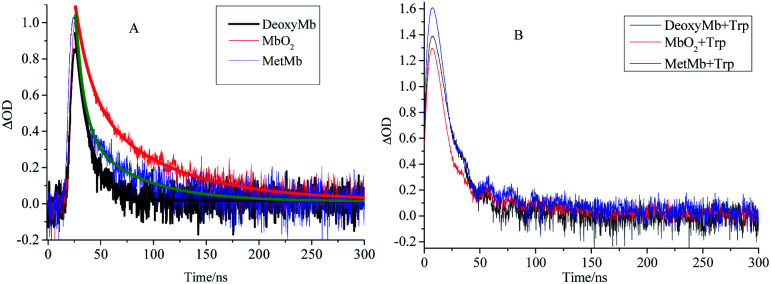
The decay kinetics curves of laser excited DeoxyMb, MbO_2_ and MetMb without (A) or with (B) Trp at the wavelengths of 430 nm, 410 nm and 405 nm, respectively.

The kinetic curves also demonstrated the efficient pathway for the energy transfer from the free excited Trp to the porphyrin. The decay kinetics curves of excited DeoxyMb (430 nm), MbO_2_ (410 nm) and MetMb (405 nm) showed diverse decay trends of the different form proteins (Fig. 2A). The decay rate of DeoxyMb and MbO_2_ were the fastest one and the slowest one among them, respectively. With the addition of tryptophan, the time decay curves of DeoxyMb, MetMb and MbO_2_ were obviously influenced (Fig. 2B). The kinetic curves were fitted with multiple exponents eqn (6) and Levenberg Marquardt eqn (7). The parameters were presented in Table S1 (ESI†). The decay process of 266 nm laser excited DeoxyMb was detected at 430 nm with a lifetime *τ* of 13.7 ns. The excited MbO_2_ has a decay process at 410 nm with *τ* = 44.78 ns. The decay lifetime of MetMb protein at 405 nm was 23.09 ns.

After adding free tryptophan, the kinetic curve of 266 nm laser excited DeoxyMb at 430 nm showed that the reaction rate slowed down with a longer decay lifetime of 20.48 ns. The fitted kinetic curve of MbO_2_ at 410 nm detected the decay lifetime with 17.24 ns, which was greatly shortened. The fitted kinetic attenuation curve of excited MetMb at 405 nm also proved a shorter decay lifetime with 18.37 ns. The lifetime data further attested that the excited tryptophan could transferred energy to heme effectively and affected the excited heme decay lifetimes.

### 3.2 UV-Vis and CD spectra

**Fig. 3:**
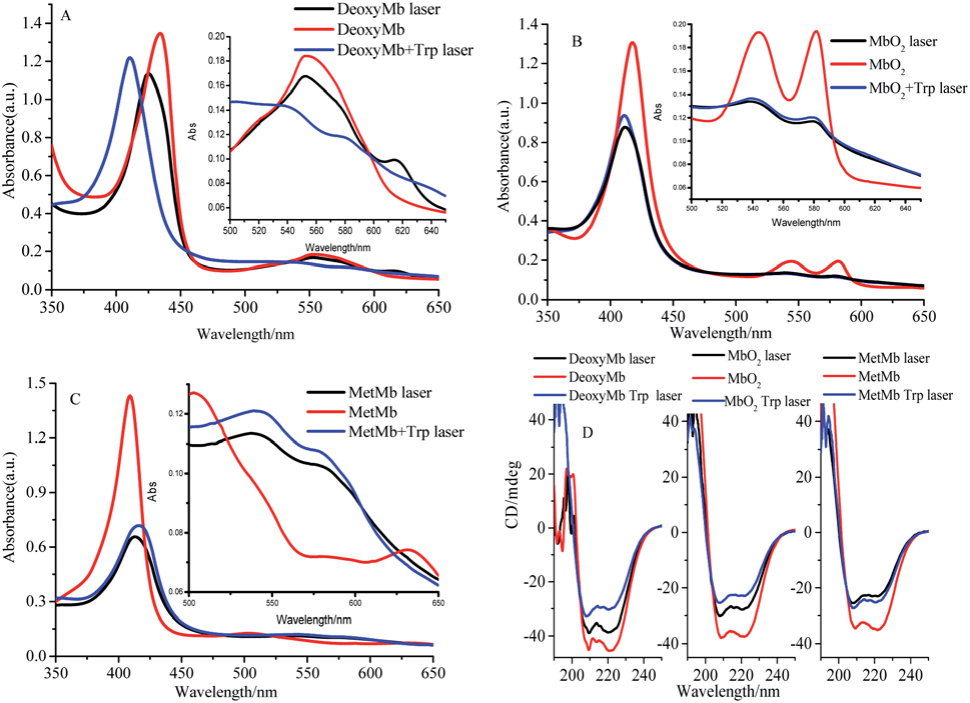
UV-Vis absorbance spectra (A–C) and CD spectra (D) of 266 nm laser photo-excited DeoxyMb, MbO_2_ and MetMb. Since the spectrum of myoglobin almost overlapped with the mixed myoglobin-Trp, the UV-Vis and CD spectra of the later were not shown.

The absorption peaks of hydrated ferric-porphyrin in MetMb located at 409, 502 and 630 nm. The absorption peaks of ferrous state MbO_2_ located at wavelengths of 420, 540 and 580 nm, while absorption peaks of the reduction state myoglobin, DeoxyMb, located at 430 and 560 nm. Since no reaction between myoglobin and free tryptophan, the UV-Vis spectra of mixed myoglobin-Trp almost shared the same line with myoglobin spectra. Thus, the overlapped spectra (UV-Vis and CD) of mixed myoglobin-Trp without laser were not exhibited in Fig. 3. The Soret absorption peak of DeoxyMb red shifted and the intensity of Q band peak at 550 nm decreased after suffering 266 nm laser flash (Fig. 3A). With the addition of tryptophan, the Q band absorption peak of DeoxyMb disappeared at 550 nm, and new absorption peaks appeared at 540 and 580 nm, which revealed that Trp could assist the heme to the photo-induced oxidation tendency. The blue shifted Soret band of MbO_2_ and the decreased peak intensity at 540 and 580 nm illustrated that 266 nm laser flash could induce the oxidation of MbO_2_ protein solution (Fig. 3B). The lower intensity and red-shifted Soret peak, the disappearance of peak at 505 nm and the new peaks at 540 and 580 nm in Q band (Fig. 3C) indicated that laser could also induce the reduction of MetMb. Fig. 3B and C manifested that free Trp have little effects on the final states of photo excited MbO_2_ and MetMb. The excited tryptophan could promote the reaction processes by intermolecular energy transfer. Interestingly, the oxidation or reduction form myoglobins reached one similar final state. So, one assumption could be safely obtained that the excited hemes in different myoglobin forms relaxed to one similar or same relatively stable intermediate state.

In proteins or polypeptide, the main chromophores groups are peptide bond in the framework of peptide chain, aromatic amino acid residue and disulfide bridge bond.^29^ The α-helix, β-sheet, β-turn and random coil structures can be detected by the far-ultraviolet CD spectrum.^27^ The secondary structures of Mbs photo-excited by 266 nm laser with free Trp were detected by ultraviolet CD spectrum as shown in Fig. 3D. Two negative peaks located at 208 nm and 222 nm were the characteristic bands of α-helix structure. Compared to the control CD spectra of three form Mbs without laser, the molar ellipticity values of the two negative peaks increased obviously but peaks kept in the same locations in the photo-excited process. The molar ellipticity values of both peaks further varied with excited free Trp after a laser-excitation. The secondary structure change of DeoxyMb was the most obvious one among them. These results further proved that the final secondary structures of the proteins were almost in a similar conformation, that is, myoglobin in three states ultimately arrived at one similar type or state.

### 3.3 Photo-induced reaction mechanism of myoglobins

**Fig. 4:**
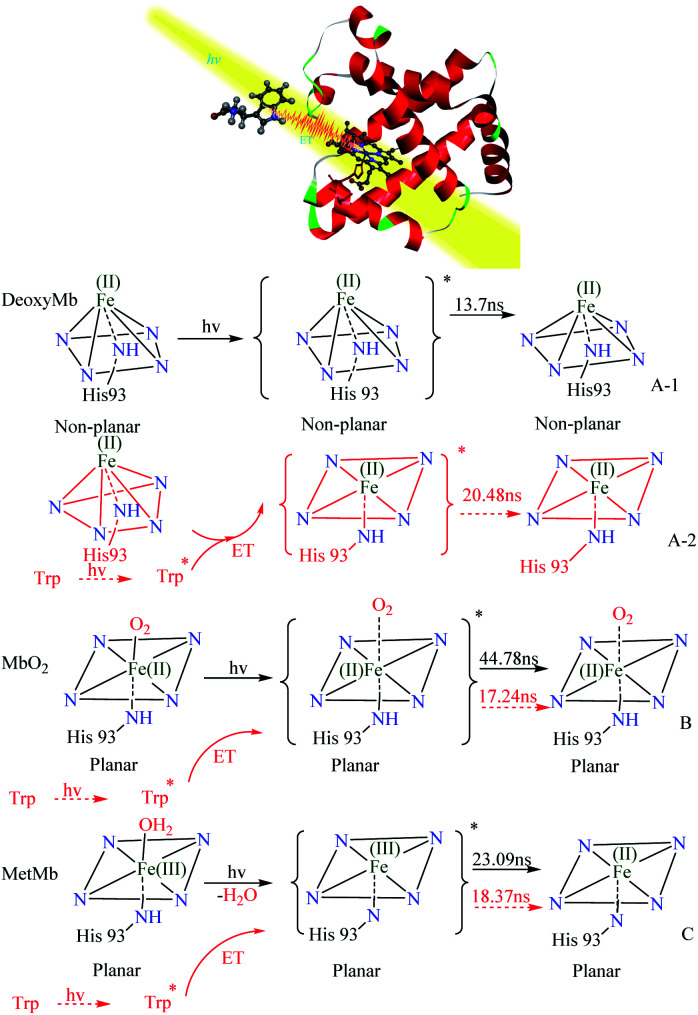
Photo-induced reaction mechanism of DeoxyMb (A), MbO_2_(B) and MetMb (C) with (black) and without (red) the effects of free Trp in the solution. ET: energy transfer.

By analyzing the kinetic curves and fitting data of laser flash photolysis, UV absorption spectrum, transient absorption spectrum and circular dichroism, we can elicit the possible photo induced reaction mechanisms of DeoxyMb, MbO_2_, MetMb. The photo direct laser excitation on porphyrin in myoglobin (PDB ID: 1DWS^30^) and energy transfer from excited tryptophan were figuratively expressed in the form of stereogram (Fig. 4). The heme of the DeoxyMb active centre was in a non-planar Fe(ii)-penta-coordinated state. Irradiated by 266 nm laser, the heme moiety in the excited state returned to the ground state by the relaxation process. The iron porphyrin transformed into a hexa-coordination state without ligand in the sixth coordination position (Fig. 4A-1). In MbO_2_, the Fe(ii) was in a hexa-coordinated state and could offer one d orbital to coordinate with the lone pair electrons of O_2_ for the carriage of oxygen *in vivo*.

When MbO_2_ suffered the 266 nm laser irradiation, the weak coordination of O_2_ toward Fe(ii) would lead to longer Fe–O coordinate bond or departure of O_2_. After the transition from excited state to a relaxed state, iron porphyrin of MbO_2_ converted into a hexa-coordination structure without ligand in the sixth coordination position (Fig. 4B). Fe(iii) in the active centre of MetMb coordinated with four ligand N atoms of porphyrin, one N atom of histidine and one O atom of water. The optical excitation on MetMb altered heme electron configuration and hexa-coordination state. One of the possible explanation was that electrons in porphyrin conjugation system transferred to the d orbital of Fe(iii), which was reduced to Fe(ii)^5,28^ and released H_2_O. Similarly, the heme moiety also converted into a stable hexa-coordination structure without ligand in the sixth coordination position (Fig. 4C).

With free tryptophan in protein solutions, the 266 nm laser not only directly excited the protein iron porphyrin, but also induced the tryptophan to various unstable excited states.^21^ The energy released from the unstable excited tryptophan transferred to different intermediates of heme, influenced iron porphyrin photo-reaction and varied the kinetic process. In DeoxyMb, the decay lifetime of iron-porphyrin from non-planar excited state to the hexa-coordination structure of ground state was 13.7 ns, while with the addition of tryptophan, the decay lifetime of excited iron-porphyrin relaxed to the hexa-coordination structure of ground state prolonged to 20.48 ns (Fig. 4A-2). With the double impacts of the direct laser and energy transferred from excited Trp, the departure or quasi-departure of O_2_ was more liable to occur in MbO_2_. Meanwhile, the transform process from ferriporphyrin to a hexa-coordinated structure without ligand in the sixth coordination position was obviously accelerated with decay lifetime decreased by 27.54 ns from 44.78 ns to 17.24 ns (Fig. 4B). After the removal of high-methionine H_2_O, porphyrin electrons are delocalized to Fe ion to form Fe(ii). As one energy transfer donor in the reaction, free excited Trp promoted the transformation of the heme moiety into a hexa-coordination structure without ligand in the sixth coordination position (Fig. 4C). Based on the ref. 20, 25, 27 and 28 and our experimental phenomena, the photo-excited endogenous free tryptophan could affect myoglobin in specific manners.

## 4. Conclusions

In summary, with the energy transferred from tryptophan, the UV absorption intensities of porphyrins in three myoglobin states increased remarkably. Combined with spectral data and kinetic data, one assumption was obtained that the excited heme in different myoglobin forms relaxed to one similar or same relatively stable intermediate state. The spectra and kinetic curves also demonstrated a more efficient pathway for the energy transfer from the free excited tryptophan to the porphyrin than that from native amino acids in the protein. Based on the above phenomena and analysis, the endogenous substances such as the tryptophan not only play the conventional function as researchers revealed, but also affect other bio-macromolecules (*e.g.* myoglobin) in various unknown styles and manners.

## Conflicts of interest

There are no conflicts to declare.

## Supplementary Material

RA-010-D0RA09341F-s001
