# Supplementary material for: Photo induced reaction of myoglobins with energy transferred from excited free tryptophan
Source: RSC Adv. 2020 Dec 9;10(71):43853–8. doi: 10.1039/d0ra09341f (PMC9058398; doi:10.1039/d0ra09341f)
Supplement: RA-010-D0RA09341F-s001 [file RA-010-D0RA09341F-s001.pdf]

Electronic Supplementary Material (ESI) for RSC Advances.
This journal is © The Royal Society of Chemistry 2020

Supplementary Information

Photo Induced Reaction of Myoglobins with Energy Transferred from Excited Free Tryptophan

Hong-Yu Cao [a,c*], Yu-Qi Ma [b], Ling-Xing Gao[b], Qian Tang[a,c], Xue-Fang Zheng [b,c*]

a College of Life Science and Biotechnology, Dalian University, Dalian 116622, China

b College of Environmental and Chemical Engineering, Dalian University, Dalian 116622, China

c Liaoning Key Laboratory of Bio-Organic Chemistry, Dalian University, Dalian 116622, China

Table S1. The parameters of kinetic curves fitted with multiple exponents by Levenberg Marquardt equation.

Protein	Wavelength (nm)	χ^2	τ (ns)	B (%)	std. Dev
DeoxyMb	430	0.695	13.70	0.93	1.53
MbO_2	410	0.907	44.78	0.90	0.65
MetMb	405	0.814	23.09	0.93	1.40
DeoxyMb+Trp	430	0.806	20.48	1.56	1.42
MbO_2+Trp	410	1.450	17.24	1.39	0.75
MetMb+Trp	405	1.330	18.37	1.73	1.07

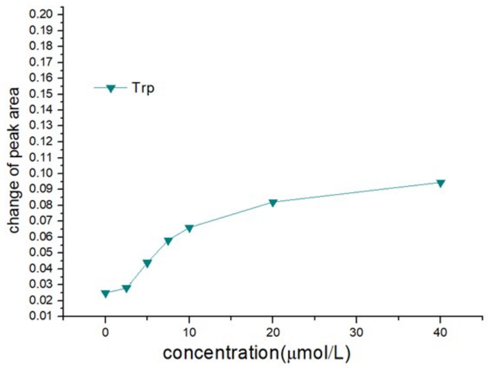

Fig. S1 The peak area changes of metMb (10^{-5}mol/L) at 544 nm after irradiation with 254 nm light in different concentration of amino acids solutions
